# PET/CT aids the staging of and radiotherapy planning for early-stage extranodal natural killer/T-cell lymphoma, nasal type: A case series

**DOI:** 10.1186/1748-717X-6-182

**Published:** 2011-12-30

**Authors:** Shannon L MacDonald, Liam Mulroy, Derek R Wilke, Steven Burrell

**Affiliations:** 1 Dalhousie University, Faculty of Medicine, 1459 Oxford Street, Dalhousie University, Halifax, Nova Scotia, B3H 4R2, Canada; 2 Department of Radiation Oncology, Nova Scotia Cancer Centre, Dickson Building, Room 2200, Main Floor, 5820 University Avenue, Halifax, Nova Scotia, B3H 1V7, Canada; 3 Department of Diagnostic Imaging, Queen Elizabeth II Health Sciences Centre, Victoria General Site, P.O. Box 9000, Halifax, Nova Scotia, B3K 6A3, Canada

**Keywords:** positron emission tomography, T-cell lymphoma, T/NK neoplasm

## Abstract

Extranodal natural killer/T-cell lymphoma (ENKTL), nasal type, is a rare form of non-Hodgkin lymphoma. Treatment of ENKTL primarily relies on radiation; thus, proper delineation of target volumes is critical. Currently, the ideal modalities for delineation of gross tumor volume for ENKTL are unknown. We describe three consecutive cases of localized ENKTL that presented to the Nova Scotia Cancer Centre in Halifax, Nova Scotia. All patients had a planning CT and MRI as well as a planning FDG-PET/CT in the radiotherapy treatment position, wearing immobilization masks. All patients received radiation alone. In two patients, PET/CT changed not only the stage, but also the target volume requiring treatment. The third patient was unable to tolerate an MRI, but was able to undergo PET/CT, which improved the accuracy of the target volume. PET/CT aided the staging of and radiotherapy planning for our patients and appears to be a promising tool in the treatment of ENKTL.

## Background

Extranodal natural killer/T-cell lymphoma (ENKTL), nasal type, has been known by various names: malignant midline reticulosis, polymorphic reticulosis, lethal midline granuloma, and angiocentric immunoproliferative lesion [1]. ENKTL often presents as a nasal mass, causing obstruction or bleeding and may elicit symptoms similar to that of a nasal sinus infection. The sites most commonly involved include the nasal cavity, paranasal sinuses, nasopharynx, hypopharynx, larynx, and tonsils [2].

ENKTL accounts for < 1% of all non-Hodgkin lymphomas in North America, making it a rare diagnosis in this population [3]. The ENKTL subtype is more common in Asia where it constitutes 6-8% of non-Hodgkin lymphomas [3,4]. The median age of diagnosis is 50 years and male-to-female sex ratio is approximately 3:1 [4]. The most common immunophenotype is CD3^-^, CD3ε^+^, CD56^+^, Epstein-Barr virus-encoded early small RNA (EBER)^+ ^[4].

Although there is no standard treatment for ENKTL, radiotherapy constitutes a significant component of its management [2,5]. This tumor type does not usually respond to anthracycline-based chemotherapy [5,6]; however, recent Phase I and Phase II trials have suggested that concurrent chemoradiotherapy using nonanthracycline-based drugs may improve outcomes in stage IE and IIE disease [6,7]. Chemotherapy post-radiation may also be beneficial [8]. Because treatment relies primarily on radiotherapy, delineating the proper target volumes for irradiation is critical.

The use of inadequately accurate imaging for radiation therapy volume delineation may lead to missed tumor or the irradiation of excessive volumes of normal tissue. Currently, the ideal modalities for delineation of the target volume for ENKTL are unknown. Computed tomography (CT) and magnetic resonance imaging (MRI) are most commonly used [2]. Positron emission tomography (PET)/CT appears to be a promising tool for the staging of and radiotherapy planning for ENKTL [9-11]. ENKTL lesions are fluorine-18 fluorodeoxyglucose (^18^F-FDG) avid [9,10], suggesting that PET/CT may be useful in detecting occult disease and involved lymph nodes [2]. Despite its promise, the utility of PET/CT in the management of ENKTL is not well described.

We present three cases of localized ENKTL and outline the utility of PET/CT in the staging and radiotherapy planning processes.

## Case Presentations

Three Caucasian patients with localized ENKTL presented to the Nova Scotia Cancer Centre in Halifax, Nova Scotia, Canada. As per institutional intensity modulated radiotherapy (IMRT) protocol, all patients had a planning CT and MRI.

These patients also underwent FDG-PET/CT in the radiotherapy treatment position, wearing immobilization masks, to help assign stage and to aid in the selection of target volumes for radiotherapy. Sixty minutes following injection with 370 MBq of ^18^FDG, imaging was performed from skull base to proximal thighs on a dedicated PET/CT scanner (GE Discovery STE16, GE HealthCare, Milwaukee).

The attending radiation oncologist delineated the gross tumor volume (GTV) based on the anatomical extent of the tumor, as determined by physical examination, as well as the planning CT and MRI. The MRI was primarily used to guide the precision of delineation. The main purpose of the PET/CT was to determine which nodes were suspicious for involvement and to guide the delineation of the extranodal component of the GTV. Standardized uptake value (SUV) thresholds were not used for tumor delineation. All patients received radiotherapy alone.

### Case 1

**Figure 1:**
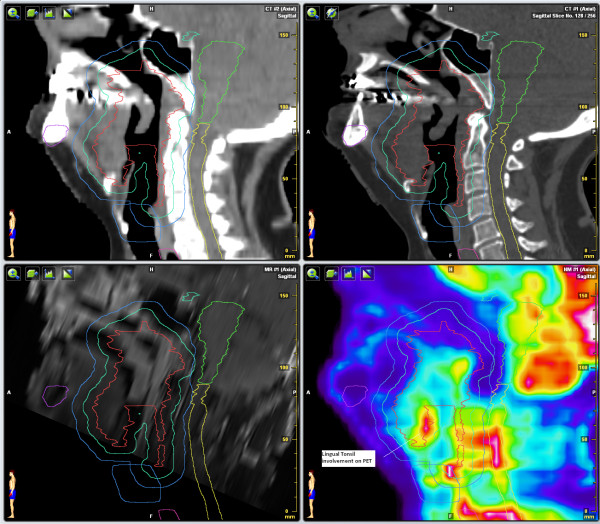
**CT (soft tissue windows and bone windows), MRI, and PET sagittal views**. Lingular tonsillar involvement is visualized on the PET, but was not detected using CT or MRI.

**Figure 2:**
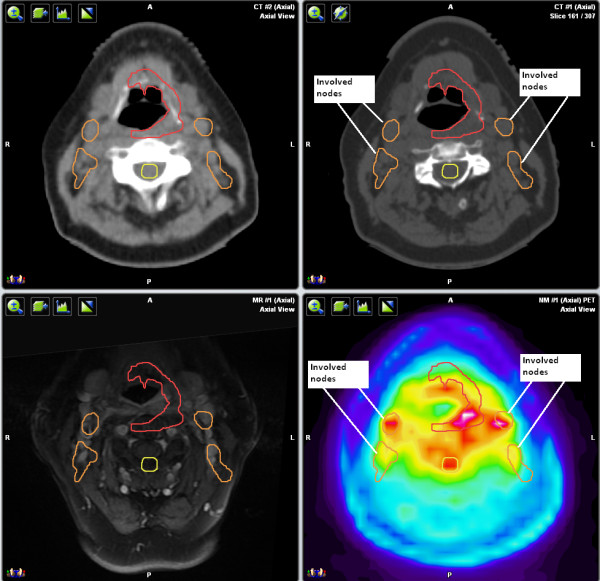
**CT, MRI, and PET axial views**. Involvement of the bilateral subcentimeter regional lymph nodes was appreciated on PET, but not on CT or MRI.

A 61-year-old woman presented with a yearlong history of chronic sinusitis and a painful ulcerative lesion involving the soft palate. A biopsy of the lesion revealed ENKTL (CD3^+^, CD56^+^, CD4^+^, CD8^+^, EBER^+^). Lactate dehydrogenase (LDH) levels were normal. Eastern cooperative oncology group (ECOG) performance status was 0. Physical examination and flexible fiberoptic nasolaryngoscopy were performed and demonstrated a tumor involving the entire soft palate, bilateral palatine tonsillar areas, and nasopharynx. Body CT, bone marrow aspirate, and bone biopsy revealed no distant dissemination. A diagnostic MRI of the neck was contraindicated as a result of the patient's implanted bladder stimulator and an MRI of the head using a head coil was performed instead. The PET/CT revealed involvement of the lingual tonsils and bilateral subcentimeter regional lymph nodes, which were not appreciated clinically or on previous imaging (Figures 1 &2).

The SUVmax of the primary lesion in the palate was 18.9 and was contiguous with the palatine tonsils. The lingual tonsils demonstrated clearly abnormal uptake and given their proximity to the primary lesion and being part of Waldeyer's Ring, it was determined that they were clinically involved by ENKTL. The SUVmax of the lymph nodes was 3.5. Although uptake in the nodes was less than that in the primary lesion, it would be underestimated given their small size. The uptake was clearly abnormally elevated and the lymph nodes were deemed likely involved.

The tumor was classified as Stage IIAE and the planned treatment volume was increased to include the lingual tonsils and regional lymph nodes. The prescribed dose to the GTV was 60 Gy/30 fractions over five weeks, six fractions per week, with one twice-a-day treatment per week. The prescribed dose to the elective nodal volume was 48 Gy/30 fractions, using a two level simultaneous integrated boost technique. The patient has been followed for 25 months without recurrence.

### Case 2

**Figure 3:**
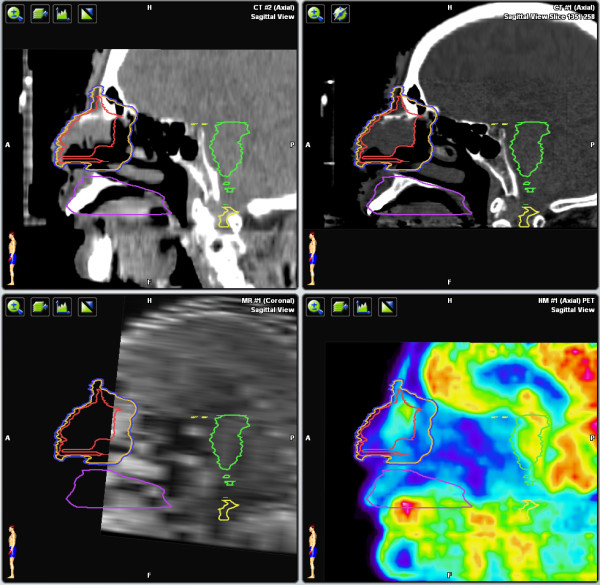
**CT, MRI, and PET sagittal views**. Patient was unable to tolerate the MRI, resulting in motion artefact.

**Figure 4:**
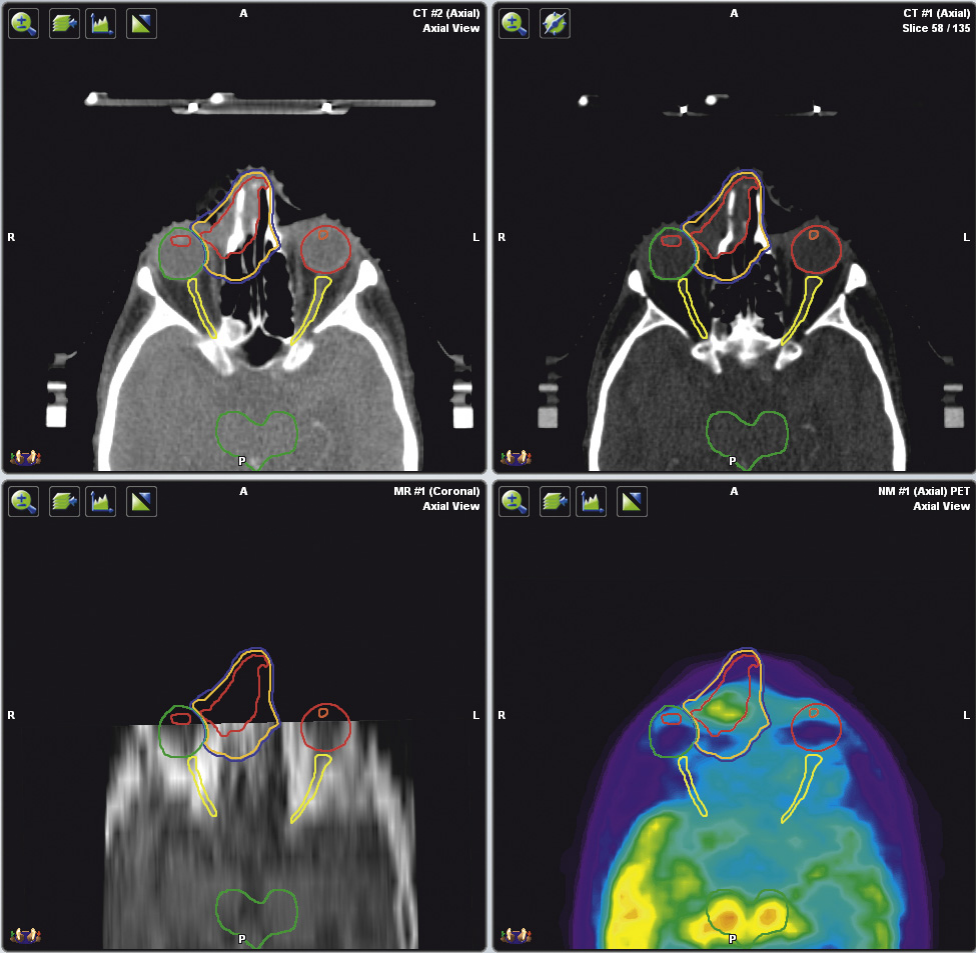
**CT, MRI, and PET axial views**. Imaging shows the tumor involves the right nasal cavity and medial orbit.

An 82-year-old man with early dementia and moderate cognitive impairment presented with an enlarging mass arising from the lateral aspect of the superior nasal cavity, invading the medial canthus of the right eye. Biopsy of the mass revealed ENKTL (CD3^+^, CD56^+^, CD4^+^, CD8^+^, EBER^+^). LDH levels were normal. ECOG performance status was 2. Physical examination and flexible fiberoptic nasolaryngoscopy revealed a 2 cm mass on the lateral aspect of the right, superior nose. The tumor did not involve the posterior nasal cavity or the pharynx. MRI was attempted, but was not tolerated. The patient was able to tolerate a PET/CT (Figures 3 &4). The PET/CT was used to outline the target volume. SUVmax of the lesion was 4.5 and the tumor was Stage IAE. The treatment plan was intended to be palliative and consisted of stereotactic IMRT, 42.5 Gy/10 fractions, using a relocatable head-frame. At the time of manuscript submission, the patient was living and had no evidence of recurrence.

### Case 3

A 49-year-old man presented with a recurrent nasal mass in 2008. Biopsy of this area showed ENKTL (CD3^+^, CD56^+^, CD4^+^, CD8^+^, EBER^+^). LDH was normal. ECOG performance status was 1. In 1999, the patient was diagnosed with Mantle cell lymphoma localized to the nasal cavity, which was treated with 35 Gy/20 fractions, using three-dimensional conformal radiotherapy. Re-analysis of the 1999 lymphoma revealed that it was also ENKTL (not Mantle cell). The patient initially declined treatment of the recurrence due to a lack of symptoms. Thirteen months later the patient agreed to re-irradiation after experiencing unilateral nasal obstruction, ocular edema, and an elevated LDH.

The long duration of disease, desire for symptom control, and the localized nature of the relapse led to the choice of re-irradiation. The patient was counseled regarding the potential risk of delayed normal tissue injury from re-radiation. Both the attending hematologist and radiation oncologist were in favor of this approach.

**Figure 5:**
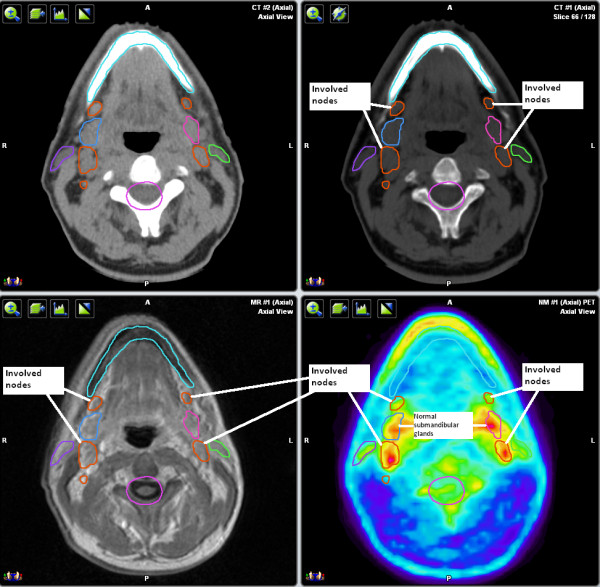
**CT, MRI, and PET axial views**. These enlarged lymph nodes were shown to be FDG-PET-avid, changing the stage from IAE to IIAE.

Clinically, the tumor was confined to the right maxillary sinus, with no regional adenopathy. A planning MRI showed enlarged regional lymph nodes and a PET/CT revealed that these nodes were FDG-PET-avid, changing the stage from IAE to IIAE (Figure 5). SUVmax of the primary lesion was 24. Level II nodes posterior to the submandibular glands were considered involved (SUV = 5) whereas involvement of the submandibular nodes was intermediate (SUV = 2.5). Given the proximity of the submandibular nodes to the level II nodes and the intermediate level of uptake on PET, our suspicion was that they were likely involved and they were targeted to receive 54 Gy/30 fractions. Because of the low degree of PET avidity of the level III nodes (SUV = 1.8) and a low level of suspicion of involvement, they were targeted to receive only 40 Gy/30 fractions, as part of the elective nodal volume.

The prescribed dose to the GTV was 54 Gy/30 fractions over five weeks, six fractions per week, with one twice-a-day treatment per week. The prescribed dose to the elective nodal volume was 40 Gy/30 fractions, using a two level simultaneous integrated boost technique. Twenty-two months after radiation, the patient has stable disease.

## Conclusions

In all three cases, PET/CT improved target and normal tissue delineation. In Case 1, PET/CT revealed the involvement of the lingual tonsils as well as bilateral subcentimeter regional lymph nodes, which were not appreciated on CT or MRI of the head. This finding changed not only the stage, but also the target volume requiring treatment. That is, without the PET/CT, part of the patient's tumor would not have been treated.

In Case 2, the patient was unable to tolerate an MRI. As tumor in the nasal cavity can be difficult to distinguish from mucous on a CT scan, the PET/CT improved the accuracy in outlining the target volume. The PET/CT was useful in determining the extent of extranodal involvement and increased the size of the GTV to be treated, compared to physical examination and the planning CT alone.

Finally, in Case 3, the MRI revealed enlarged lymph nodes that were not initially thought to be involved in the disease process, but were found to be enlarged by tumor on PET/CT. This finding changed the tumor stage as well as the treatment target volume.

As stated previously, radiotherapy plays an important role in the management of ENKTL. However, the failure rate for radiation alone is between 25% and 30% for early stage disease; the presence of occult disease most likely accounts for a portion of this failure rate [8]. In order to address distant occult disease, the addition of chemotherapy to radiation treatment has been explored. Recent evidence suggests that concurrent chemotherapy or chemotherapy post-radiation may be required in high-risk, early-stage disease [8].

Due to the high reliance on radiotherapy for the treatment of ENKTL, proper delineation of the target volume is essential. MRI is often used for radiotherapy planning, but it requires a cooperative patient and has several contraindications. A diagnostic MRI of the neck was contraindicated in one our patients and was not tolerated in another. Nonetheless, most patients are able to tolerate an MRI.

Even when MRI is possible, determining whether there is lymph node involvement can be difficult. In Case 3, there was uncertainty as to whether the lymph nodes were involved based on CT and MRI. Lymph node size is often used to determine whether metastases are present; however, lymph nodes can be enlarged as a result of benign processes such as inflammation and infection [12]. Conversely, small volume disease can be present, but not visible on CT or MRI [12]. PET/CT was more definitive in distinguishing malignant from normal tissue in this case and changed the stage as well as the treatment plan to involve irradiation of the nodes.

Few studies have examined the utility of PET/CT in the management of ENKTL; however, the data that do exist are promising. Most identified ENKTL lesions are FDG-avid [9,13,14]. PET/CT results may be less reliable for hepatic lesions and bone marrow involvement; Karantanis et al. described ten patients, of which one with ENKTL and one with NK-cell lineage lymphoproliferative disorder had hepatic metastases and bone marrow involvement, respectively, that were not identified on PET/CT [15].

Others have reported the utility of PET/CT in detecting involved tissues not identified by CT and/or MRI [9,16]. Berk et al. described how PET/CT detected involved regional and distal lymph nodes not appreciated using CT in a patient with ENKTL [16] and Kako et al. reported that out of eight patients, PET/CT identified three involved areas not appreciated by conventional methods. Unfortunately, the involved areas were not defined in the Kako et al. paper [9].

Abnormal FDG uptake is typically defined as an increase in background activity in surrounding tissue that is unrelated to physiologic sites of uptake or excretion [9,14,15]. One study has reported increased FDG uptake in the soft palate (mean SUV 3.31), palatine tonsils (mean SUV 3.48), and lingual tonsils (mean SUV 3.11) of patients with no known head and neck malignancy [17]. This finding suggests the possibility of obtaining false-positive results when using PET/CT in the head and neck area.

The incidence of false-positive results using PET/CT in the management of ENKTL is unknown. Karantanis et al. reported no false-positive results in their ten patients. Kako et al. described one patient (out of eight) with ENKTL that showed bone marrow involvement on PET/CT that was negative on biopsy and aspiration [9].

In our patients, the areas deemed positive were interpreted by the reporting nuclear medicine physician as *abnormal *and *probably *involved based on experience. Definitive tissue confirmation is generally not practical or warranted for all lesions. However, since we did not biopsy the tissues that were FDG-avid, but not appreciated on CT and/or MRI, we cannot exclude the possibility that the PET/CT results represented false-positive results.

In all three cases, PET/CT improved target volume delineation. We believe that PET/CT is a promising tool that may be useful in the staging process and delineation of treatment target volumes in patients who will receive radiotherapy for ENKTL. Further validation is required in a larger cohort of patients.

## Consent

Written informed consent was obtained from the patients for publication of this case series. Copies of the written consents are available for review by the Editor-in-Chief of this journal.

## Competing interests

The authors declare that they have no competing interests.

## Authors' contributions

SLM made substantial contributions to the conception, design, and drafting of the manuscript. LM contributed significantly to data acquisition and revision of the manuscript. DRW contributed significantly to data acquisition and drafting/revision of the manuscript. SB contributed significantly to data acquisition and revision of the manuscript. All authors read and approved the final manuscript.
